# Brain electrical traits of logical validity

**DOI:** 10.1038/s41598-021-87191-1

**Published:** 2021-04-12

**Authors:** Francisco Salto, Carmen Requena, Paula Álvarez-Merino, Luís F. Antón-Toro, Fernando Maestú

**Affiliations:** 1 Universidad de León, León, Spain; 2 Universidad of Valladolid, Valladolid, Spain; 3 Laboratory of Cognitive and Computational Neuroscience, Universidad Complutense/Universidad Politécnica, Madrid, Spain

**Keywords:** Neuroscience, Cognitive neuroscience, Cognitive control, Intelligence, Problem solving

## Abstract

Neuroscience has studied deductive reasoning over the last 20 years under the assumption that deductive inferences are not only de jure but also de facto distinct from other forms of inference. The objective of this research is to verify if logically valid deductions leave any cerebral electrical trait that is distinct from the trait left by non-valid deductions. 23 subjects with an average age of 20.35 years were registered with MEG and placed into a two conditions paradigm (100 trials for each condition) which each presented the exact same relational complexity (same variables and content) but had distinct logical complexity. Both conditions show the same electromagnetic components (P3, N4) in the early temporal window (250–525 ms) and P6 in the late temporal window (500–775 ms). The significant activity in both valid and invalid conditions is found in sensors from medial prefrontal regions, probably corresponding to the ACC or to the medial prefrontal cortex. The amplitude and intensity of valid deductions is significantly lower in both temporal windows (p = 0.0003). The reaction time was 54.37% slower in the valid condition. Validity leaves a minimal but measurable hypoactive electrical trait in brain processing. The minor electrical demand is attributable to the recursive and automatable character of valid deductions, suggesting a physical indicator of computational deductive properties. It is hypothesized that all valid deductions are recursive and hypoactive.

## Introduction

Deductive inferences and deductive arguments are distinct phenomena. While inferences are cortical time-consuming events^1^, arguments are abstract relations among propositions or probabilities^2^. Inferences and arguments both have the same < premises, conclusions > structure but have crucial differences. Inferences happen when they are drawn or made, while arguments don’t need to be factual. For example, inferences have a finite temporal duration, but an argument may contain infinite premises. The same exact argument can prove the irrationality of √2 using a drawing in the sand or an algebraical formulation, but visual and propositional inferences can be quite different processes. Assuming there is a clear difference between deductive and non-deductive *arguments*^3^, the objective of this paper is to determine if there is also a neural difference between these *inferences*, and to this end, logical validity is electrically studied. Both phenomena (arguments and inferences) are often fruitfully mixed in deductive reasoning as a modest but essential resource in science and everyday life. Most neuroscientific research has taken for granted that since there are normative distinctive features of deductive arguments, there are corresponding deductive inferences. But in fact, it is not known if there are specifically deductive cortical activities or if instead the deductive character of inferences is a normative feature that supervenes on neural processes. For example, when we deduce that a man is poor from seeing him asking for money, or when we validly deduce that there are infinite prime numbers, is there some kind of cortical difference between valid and invalid deductions? Are there factual marks of valid deductions as time-consuming events, or, on the other hand, is deduction a way of justifying inferences? This is a crucial open question involving the relationship between physical and computational Marr levels for which the neuroscience of deductive rationality is trying to offer an answer. As an illustration, see the contrasting arguments by Evans and Over^3^ [“since induction and deduction serve quite different purposes, it is likely that they have correspondingly different psychological mechanisms”] and Oaksford^4^ (“tasks are not deductive in and of themselves. What function a task engages is determined by the empirically most adequate computational level theory of that task”]. Our focus is finding neural evidence of deductivity in logically valid inferences^5,6^, which are those whose outputs systematically preserve the truth of their inputs.

In the last 20 years, neuroscience has made significant contributions to the study of the neural correlates of deduction under the a priori assumption that deductive phenomena are factive (that is to say, they are factual; in other words, they correspond to genuine neural processes). Cornerstones of this research are Goel’s^7^ and Prado’s^8–10^ meta-analyses and reviews; they show how distinct neural networks explain data variance between propositional, categorical and relational inferences. Notice how different arguments determine different inferences in spatial/propositional circuits, but on the other hand the literature has identified a bunch of multimodal operators for both spatial and linguistic format^11,12^. Another fundamental propositional result is the neural re-processing, or double processing, of logical connectives^13^ related to cognitive inhibition^14^ and deductive training^15,16^. The neural study of propositional inferences has focused on premise-conclusion integration, which consists of premises and conclusions sharing literal variables^17–21^. For example, the following inference^17^ is integrable: from {There is a square; If there is a square, then there is a rhombus} deduce {There is a rhombus}, but the following inference is not integrable: from {There is a square; If there is a hexagon, then there is a triangle} deduce {There is a rhombus}. “Square” appears in the conditional and in the antecedent, thus facilitating the MP inference. Through both linguistic and visual formats, research has proven the neural impact of premise integration and has located its processing in typically semantical areas of the brain. Moreover, spatial (fMRI, PET)^1,17–20^ and temporal (MEG, EEG)^21–24^ research has tied propositional deductions to the integration of premises. The literature shows that both valid and non-valid propositional reasoning involves left frontoparietal circuits related to linguistic areas. Reverberi^18,20^ has showed that in simple valid deductive inferences (such as Modus Ponens and Disjunctive Syllogism), neural processing is determined by its relational complexity, which is the number of variables appearing in the deductive task and the arity and number of functional arguments in their relations^25^. For example, in sudoku games or Latin square tasks, the relational complexity is determined by the size of the matrix and the number of rows and columns that need to be simultaneously considered. From this integration-based approach to deduction, we may interpret that valid and invalid deduction develop neurally over the same substrate, which basically depends on the information’s semantic content and not on its logical structure. This is not an isolated cognitive discovery; instead it is a consistent trend correcting previous formalistic approaches to deduction, as shown by Evans et al.^26^. However, the perspective based on premise integration excludes a wide family of deductive inferences which are valid but not integrable, and which are in fact eventually used as a control or baseline in deductive reasoning experiments because their conclusions are not integrable with their premises. In this regard, integration does not offer a single neural support for deductive and non-deductive inferences (since valid deductive inferences are excluded).

A second minoritarian research line for the study of the neural correlates of propositional deduction has focused on complexity and not on integration. Logical complexity is the number of occurrences of logical operators in a cognitive task. For example^27^, the inference from {**If** the block is **either** round or large, then it is **not** blue; The block is round} to {The block is **not** blue} has 4 instances of logical operators; two of them are dyadic (if… then, either… or), and two are monadic (not). The conclusion of the argument has a logical complexity of 1, and its minor premise has 0 logical complexity. The strategy of Osherson^28^, Monti^12,27,29,30^ and Coetzee^31,32^ was to study the neural effect of amplifying logical complexity in reasoning tasks while maintaining the same relational complexity. In this regard, the cerebral correlates of logical complexity are experimentally identified and dissociated from semantic content processing. The research methodology has been progressively refined over the years, and as a result, an extended sequence of studies has shown the involvement of specifically deductive “core” frontal areas (including both the mesial BA8 and left rostrolateral prefrontal cortex in BA10) which do not coincide with the linguistic areas identified by the integrational perspective.

There are logical and probabilistic measures of validity which are complete, precise and decidable, but they are abstract computational procedures which do not directly correspond to any spatio-temporal processes in the brain. Cognitive science has searched for factive deductivity measures which were experimentally viable, as the early work of Rips^33^ and Wilhelm^34^ show. In this regard, the proposal by Rotello and Heit^35,36^ contrasting the impact of validity with that of the number of words in deductive inferences is remarkable. In the field of cerebral research, two indexes have been proposed and employed by Reverberi^18,19^ associating physical magnitudes with logical validity. Other researchers^34,37^ have systematized deductivity measures in psychological and neural contexts. The new experimental paradigm presented in this paper avoids any need to measure relational complexity because it remains fixed or unaltered while logical complexity is measured in the usual way. Two methodological novelties are introduced. First, the strategy of Osherson and Monti is altered to avoid recalcitrant increases in logical complexity and second, we chose not to study what Reverberi calls “the instant of deduction”, and instead of comparing specific inferences sequentially analyzed in premises and conclusions, we evaluate the cerebral processing of valid inferences in toto, irrespective of the specific connectives or operators used in any trial.

The objective of this research is to identify features or brain activity traits of logically valid deductive inferences distinct from those of logically invalid inferences. If these features are identified, then it is feasible to acknowledge factive properties (in this case neuroelectrical properties) of valid deduction. If they are not found, this would be a clue that logical validity is a purely normative phenomenon. Considering the limitations identified in the state of the art, this research: (i) does not a priori assume the existence of factive differences between valid and non-valid inferences, (ii) does not dismiss non-integrable content-independent deductive inferences, (iii) does not exacerbate the logical complexity of tasks to electrically measure the difference between the neural processing of logical and relational complexity. Moreover, the work employs a new experimental paradigm in which two conditions (valid/non-valid) with exactly the same relational complexity and different logical complexity are compared.

## Method

### Participants

Twenty-three young right-handed subjects (12 males and 11 females) make up the MEG database. All participants were recruited during the months of May and June 2019 from the Complutense University of Madrid and the University of Villanueva during May and June 2020 and received academic credit for their participation. After initial signal-quality checks, the data of three participants were removed from our sample due to bad data quality. The final dataset was composed of 20 subjects (10 males and 10 females), with a mean age of 20.35 years (SD =  ± 3.23). Participants did not report any significant neurological or psychopathological conditions or any psychoactive drug intake during MEG recordings. Each participant went through two experimental tasks sequentially. First, they performed a LOGICALLY INVALID paradigm task (the control task), whereupon they performed a LOGICALLY VALID paradigm. The responding hand for each condition was counterbalanced across subjects. All participants signed an informed consent form before their participation in this study, following the guidelines in the Declaration of Helsinki. The study was conducted in accordance with the guidelines of Research Ethics of RCEUE, (Red de Comités de Ética de la Investigación de Universidades Públicas Españolas) (Ethics Committees Network of Spanish Public Universities) and was approved by the “Comité de Ética de la Universidad de León” (Ethics Committee of the University of León), dated 11-06-2019 and registered as “ETICA-ULE-O181-2019”.

### Stimuli

The items in the study were trios of cards from the game SET (Set Enterprise, 2019). Each card has a variation of the following four features: figure (diamond, ovoid, squiggle), color (green, red, black), cardinality (1 or 2), filling (filled, empty). None of the participants was familiar with the game or its rules. The experiment included the same 200 randomly ordered trials in both conditions. Presenting the same stimuli ensures that the relational complexity of both conditions is exactly the same; that is, they include the same set of cards and are described with the same nomenclature and lexical card descriptions. On the other hand, instructions for the valid condition (SET definition and logical constants) ensure that the valid condition has a measurable logical complexity.

**Figure 1:**
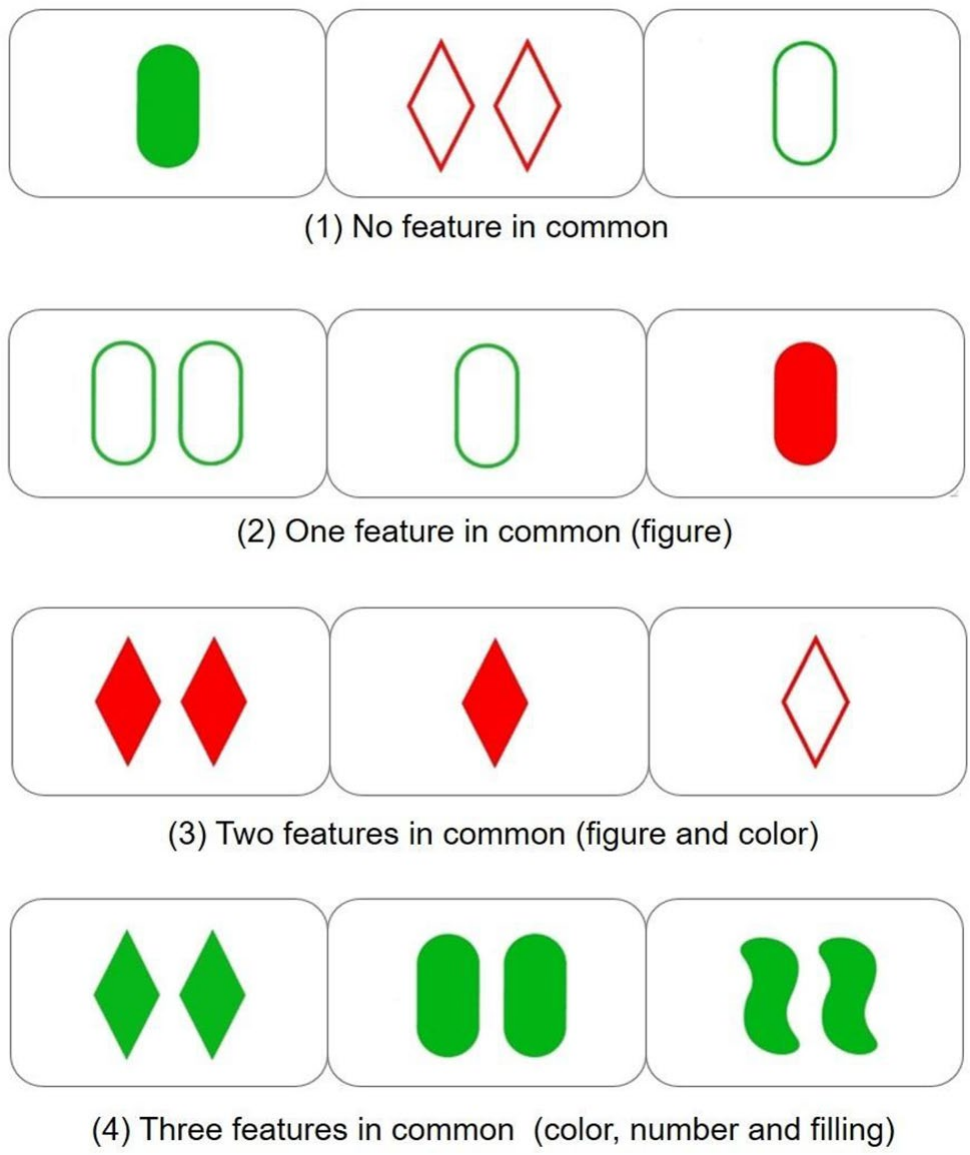
Example of items of SET in both conditions.

Any given trio of cards either does or does not share the same features, figure, color, number and filling. The following are examples of trios which don’t share any features (case 1), share one feature (case 2), share two features (case 3), or share three features (case 4) (see Fig. 1).

### Time chart

**Figure 2:**
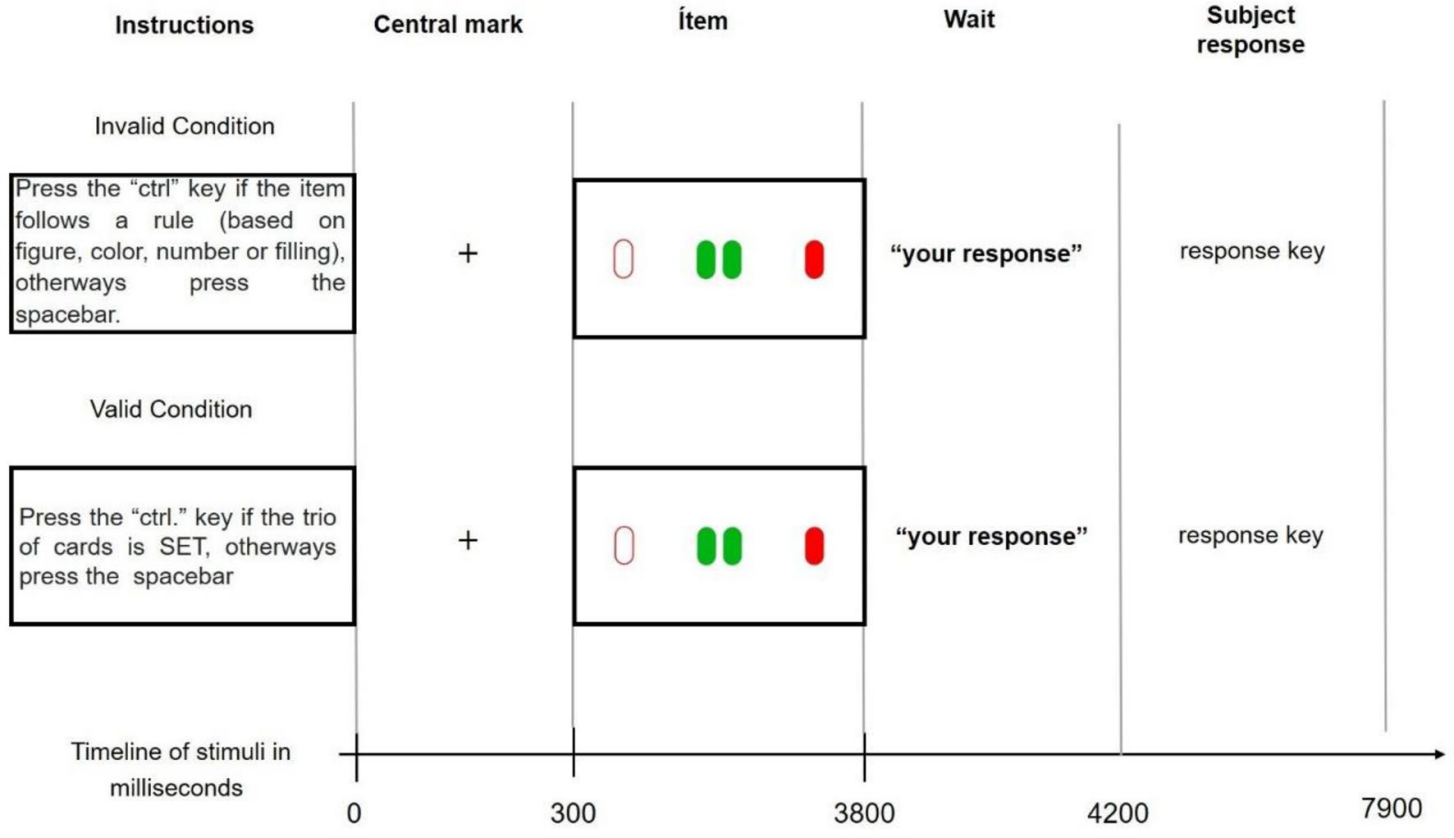
Timeline of stimuli in milliseconds.

The beginning of the trial was signaled by an asterisk (+) presented in the center of the screen for 300 ms, which was then followed by the appearance of the items on the screen for an additional 3500 ms. at which point the items disappeared and the central dot reappeared for 400 ms. Finally, participants were asked to respond quickly for 3700 ms. The time chart is presented in Fig. 2.

### Experimental design paradigm task

The experiment contrasts two inferential tasks which contain the exact same stimuli, meaning the same relational variables with the same contents and properties. The logically valid task includes explicit deductive rules as instructions, while the logically invalid task has no valid deductive rules to follow.

In the logically invalid task, the subject does not receive any specific deductive rule; instead, they are shown a set of visual stimuli (SET cards) and informed about the cards’ features (figure, color, number, filling). The instructions for the logical invalid task were: “If an item follows a rule based on color, figure, number or filling, press the ‘Ctrl’ key, otherwise press the space-bar”. In this way, the invalid condition acts as a control of the valid condition, since the subjects face a rule search task with the card features already given by the premises.

In the logically valid task, the subjects must validly deduce their answer given the definition of what makes up a SET and after being shown a trio of cards. The logical properties of a SET allow one to deductively determine if any given trio is or is not a SET exclusively applying tools from elementary first order logic. Any given trio is a SET if all the cards have two or more properties in common. The deductive instruction is: “Press the ‘Ctrl’ key if the presented trio is a SET, otherwise press the space-bar”.

The logical structure of the definition of SET is informally given by the following SET definition:

{[same filling AND (same form OR same number OR same color)] OR [same form AND (same filling OR same number OR same color)] OR [same number AND (same form OR same filling OR same color)] OR [same color AND (same form OR same number OR same filling)]} ↔ SET.

This definition shows that inferences in the valid condition are also p-valid (since they are logically valid and only contain truth functional operators, see^6,38^) and they help the reader to explicitly follow Modus Ponens and Modus Tollens applications.

The deduction of the answer (is a SET/is not a SET) is stated without any previous training. It is an integrable inference in all trials and crucially depends on the definition of SET, hence excluding any non-deductive heuristics. It is essential to recognize that the valid task, even if it is simple, has a non-null logical complexity. The experimental design presents two tasks with the same relational complexity but distinct logical complexity. The design does not allow the researchers to describe the precise inference pattern followed by any agent in any trial. For cases (3) and (4), positive propositional inferences are enough, particularly connectives and the Modus Ponens rule (deduce B from {A, if A then B}). Cases (1) and (2) can be negatively treated with connectives and the Modus Tollens rule (deduce not A from {not B, if A then B}). The point of the experiment is not to follow the neural processing of a specific pattern, but to study any deductively valid inference. This task is ecological and user-friendly, since it is inspired and presented as a game.

The experiment is programmed and administered using E-PRIME software. The screen CRT has an actualization frequency of 60 Hz, and a resolution of 1024 × 768 pixels. Items in all stimuli are presented against a black background. In both tasks, the index fingers of both hands press keys on a computer keyboard to answer. Participants sit 60 cm in front of the screen in a quiet dimly lit environment.

### MEG recordings

MEG data was acquired via a 306-channel Elekta Neuromag system located in the Center for Biomedical Technology (Madrid, Spain), using an online anti-alias filter between 0.1 and 330 Hz and a 1000 Hz sampling rate. Environmental noise was reduced using an offline signal space separation method^39^, and subject movements were compensated using the same algorithm. The head shape of the participants was acquired using a three-dimensional Fastrak digitizer (Polhemus, Colchester, Vermont), and three fiducial points (nasion and left and right pre-auricular points) were used as landmarks. Four Head Position Indicator (HPI) coils were placed on the participants’ scalp (two on the forehead and two on the mastoids) and their position was recorded.

For the pre-processing pipeline, we used the FieldTrip package^40^ to automatically detect ocular, cardiac and muscle artifacts, which were subject to validation by an MEG expert. Finally, we used an independent component analysis based on SOBI^41^ to remove eye-blink and cardiac activity. The data was segmented according to the task events into 1500 ms trials (500 ms of baseline and 1000 ms of task-related data), and trials marked as containing artifacts were discarded from subsequent analysis. Finally, trials with a response time (i.e., time elapsed between the stimulus onset and the response) of less than 1 s were discarded to minimize the influence of motor response over interest time windows.

## Results

### Behavioral data

RT measure was obtained for each subject and each condition. It was calculated from the onset of the cards trio, within a temporal window of 1 s. Higher RTs were observed in response to the logically Valid condition (Table 1), with a significant main effect p = 0.00012 in the t-test (valid-invalid) analysis for related samples.

**Table 1:** Descriptive data of RT of the conditions.

RTs	Mean	Median	SD
Valid condition	2667.89	2273.27	1766.47
Invalid condition	1450.53	1160.22	783.13

In the logically valid condition, the subjects answered correctly 92.44% of the trials and incorrectly for the other 7.66%. The incorrect responses were not considered in the analysis. In the logically invalid condition, there were no evaluable correct or incorrect answers. However, 74.51% of the subjects answered “no” to the 33% of the trials without any feature in common (figure, color, number, filling). Therefore, it seems that the subjects understood the most obvious interpretation of the invalid task, and most of their answers were not arbitrary.

### MEG ERF analysis

**Figure 3:**
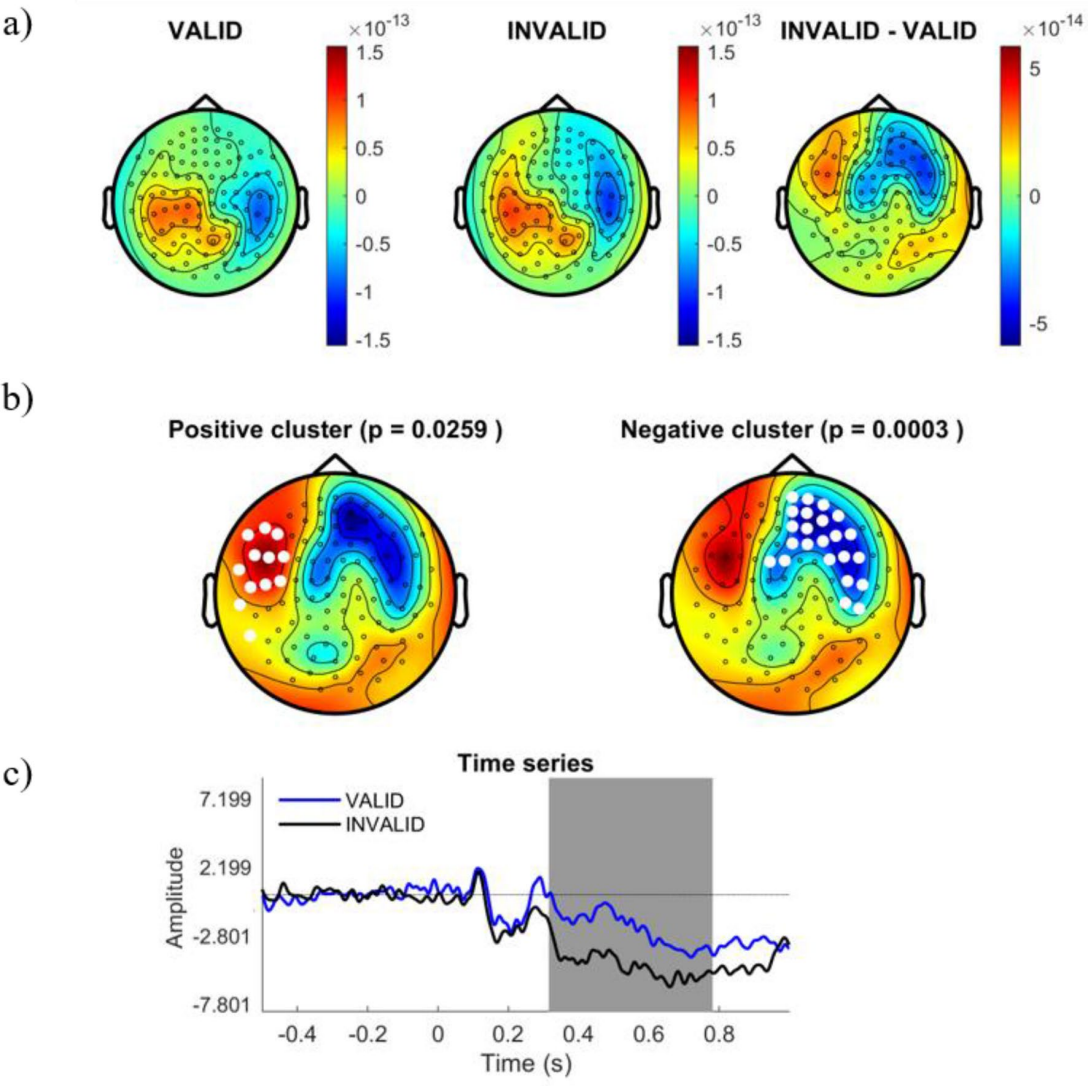
Represents the distribution of cortical activity from 310 to 780 ms. (**a**) Shows topographical maps of amplitudes for each condition (left and central map) and their arithmetic subtraction (right map). (**b**) Represents statistical differences (T values) between conditions. Left, positive cluster, and right, negative cluster. White dots represent sensors that showed significant differences during time-window. (**c**) Shows the whole time-series for both conditions (blue = VALID; black = INVALID). Grey mask represents time-slice of samples with significant results.

**Figure 4:**
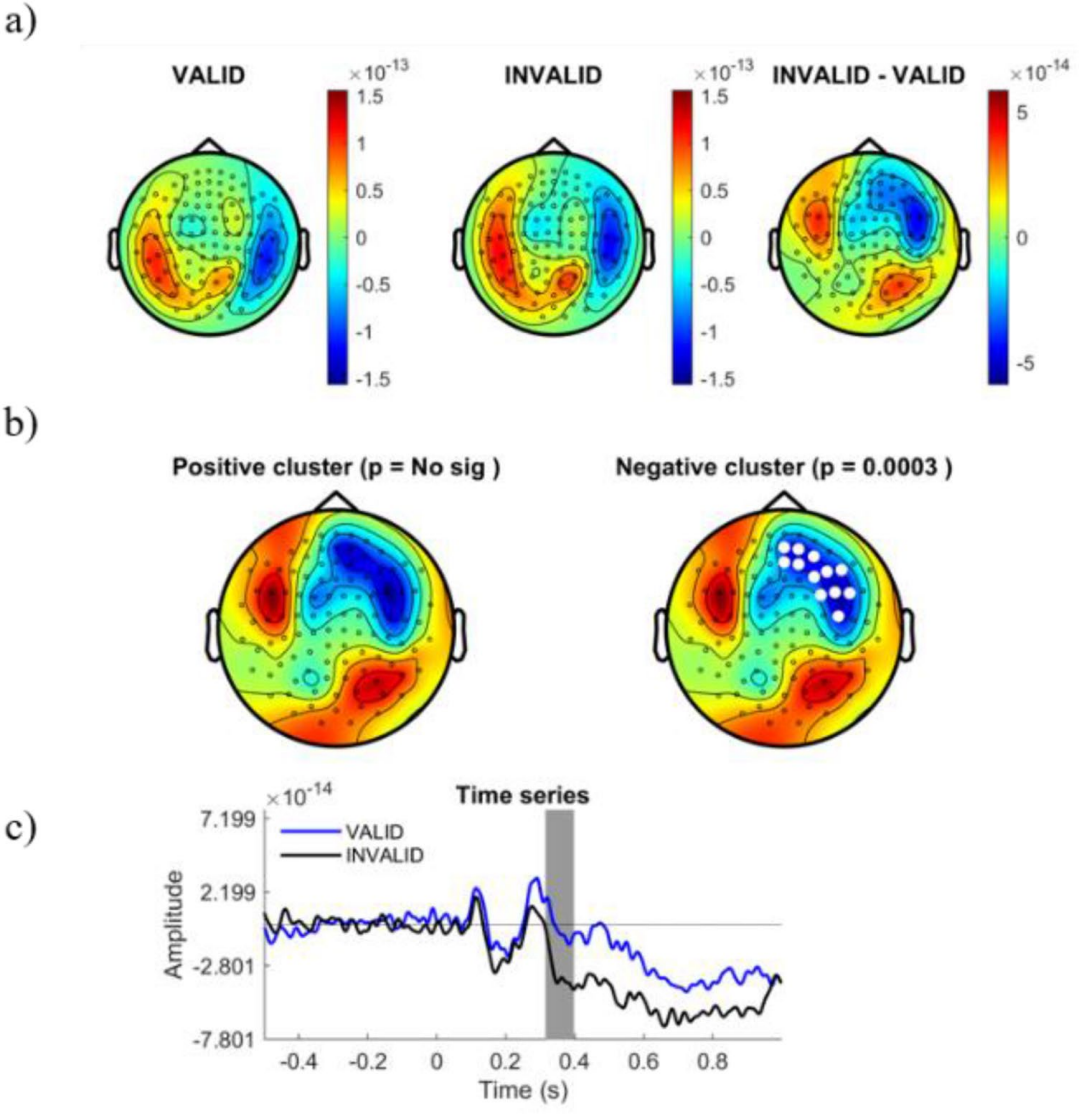
Represents the distribution of cortical activity in a time-slice from 250 to 400 ms. (**a**) Shows topographical maps of amplitudes for each condition (left and central map) and their arithmetic subtraction (right map). (**b**) Represents statistical differences (T values) between conditions. Left, positive cluster, and right, negative cluster. White dots represent sensors that showed significant differences during time-window. (**c**) Shows the whole time-series for both conditions (blue = VALID; black = INVALID). Grey mask represents time-slice of samples with significant results.

**Figure 5:**
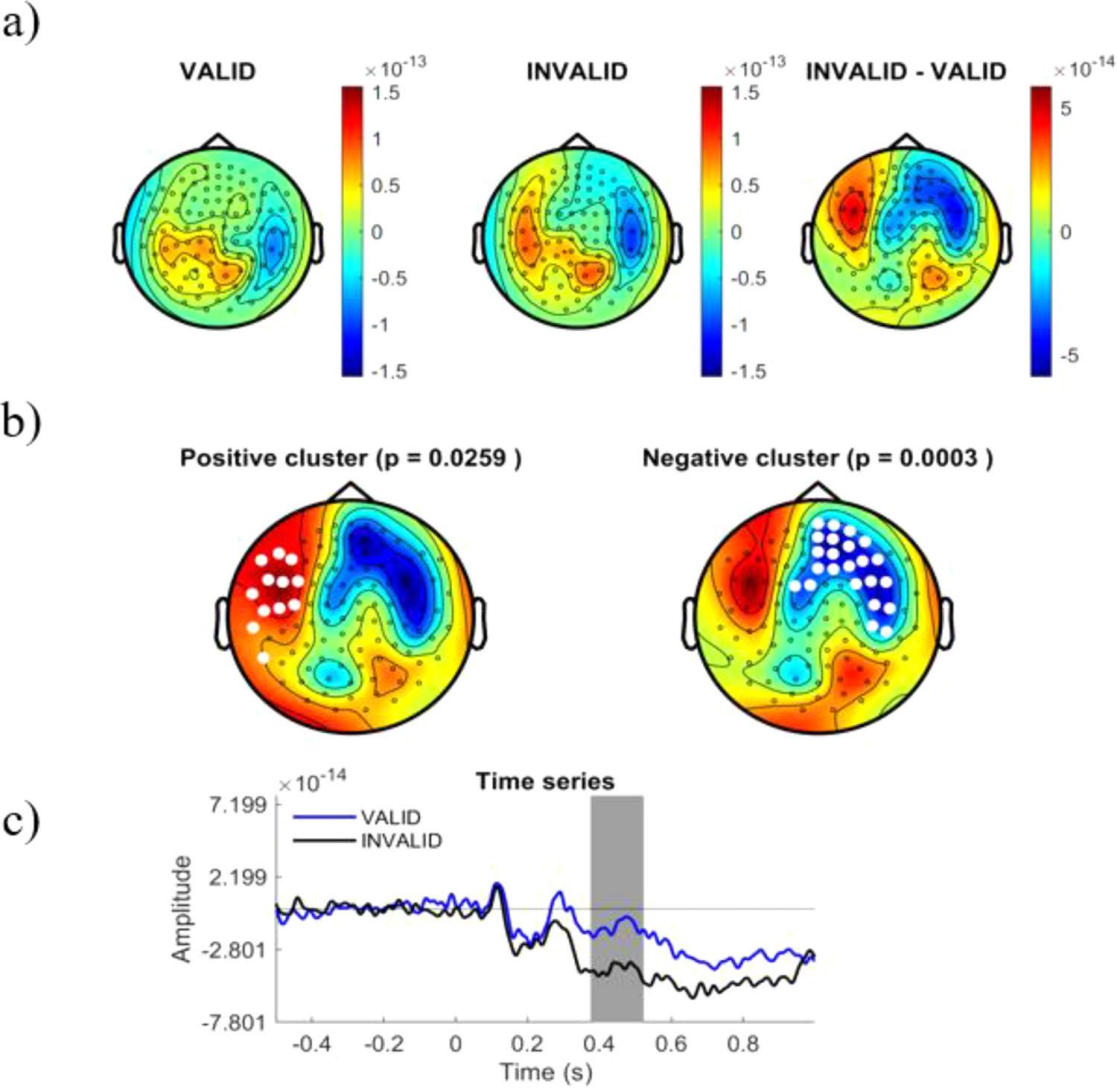
Represents the distribution of cortical activity in a time-slice from 375 to 525 ms. (**a**) Shows topographical maps of amplitudes for each condition (left and central map) and their arithmetic subtraction (right map). (**b**) Represents statistical differences (T values) between conditions. Left, positive cluster, and right, negative cluster. White dots represent sensors that showed significant differences during time-window. (**c**) Shows the whole time-series for both conditions (blue = VALID; black = INVALID). Grey mask represents time-slice of samples with significant results.

**Figure 6:**
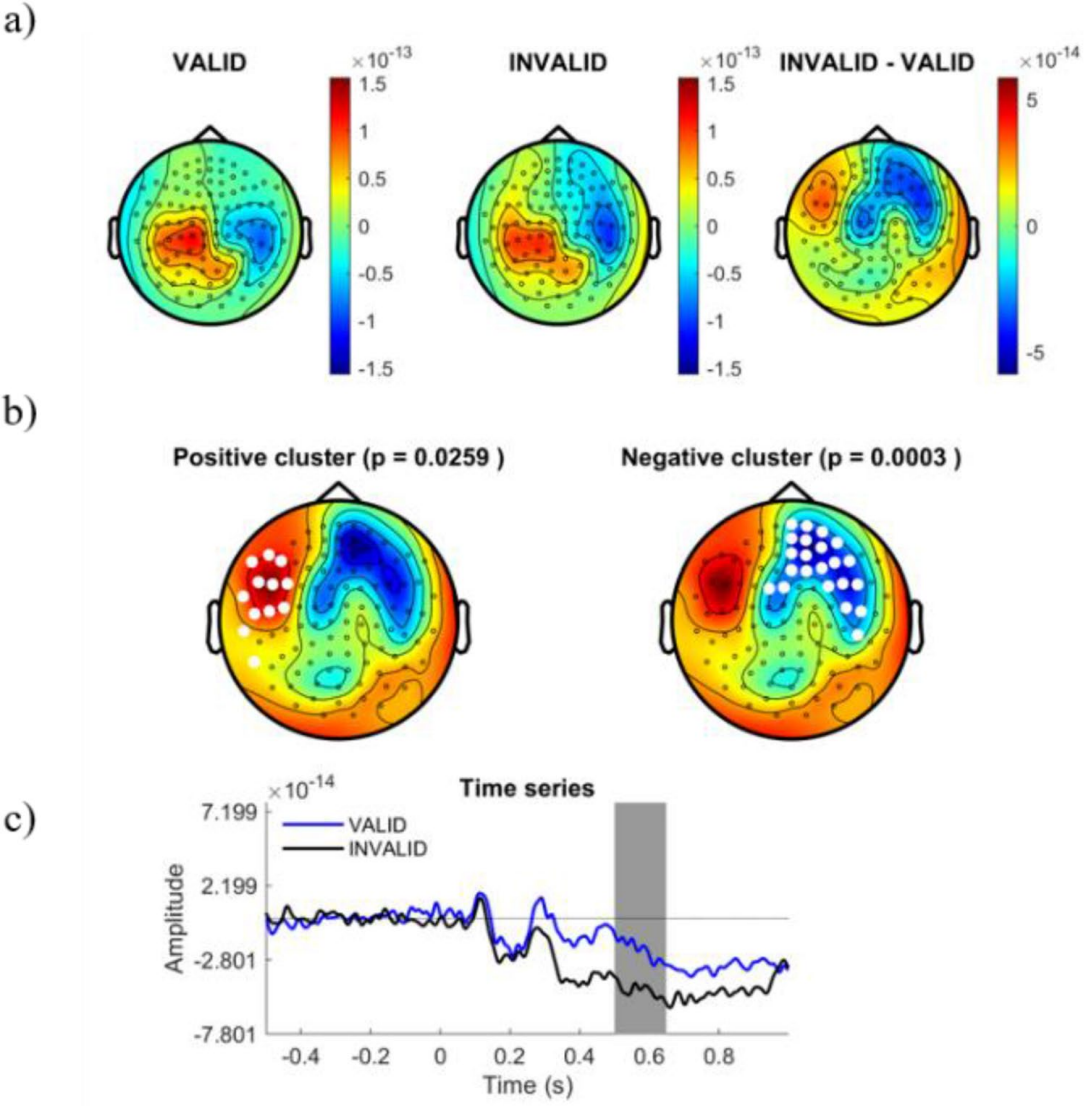
Represents the distribution of cortical activity in a time-slice from 500 to 650 ms. (**a**) Shows topographical maps of amplitudes for each condition (left and central map) and their arithmetic subtraction (right map). (**b**) Represents statistical differences (T values) between conditions. Left, positive cluster, and right, negative cluster. White dots represent sensors that showed significant differences during time-window. (**c**) Shows the whole time-series for both conditions (blue = VALID; black = INVALID). Grey mask represents time-slice of samples with significant results.

**Figure 7:**
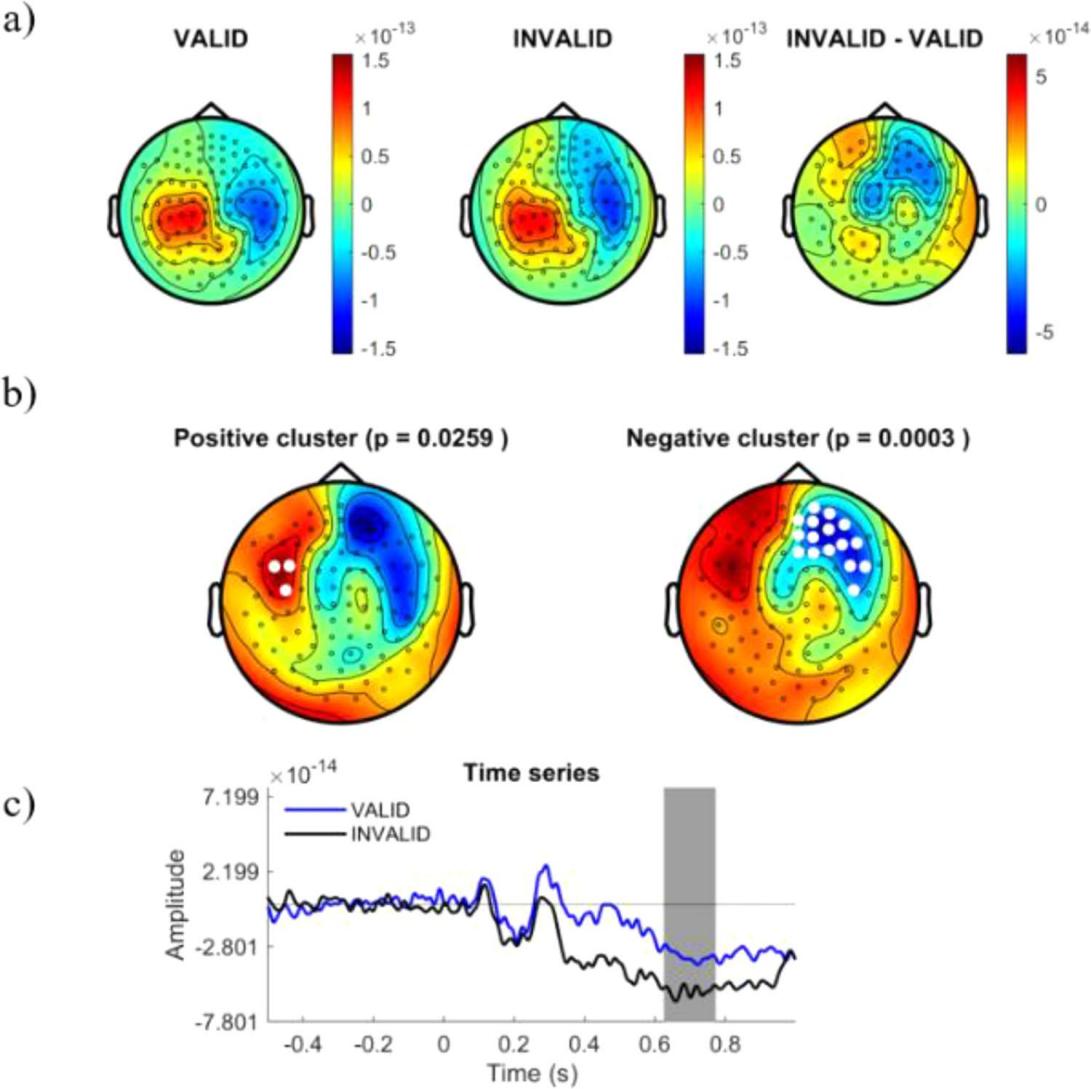
Represents the distribution of cortical activity in a time-slice from 625 to 775 ms. (**a**) Shows topographical maps of amplitudes for each condition (left and central map) and their arithmetic subtraction (right map). (**b**) Represents statistical differences (T values) between conditions. Left, positive cluster, and right, negative cluster. White dots represent sensors that showed significant differences during time-window. (**c**) Shows the whole time-series for both conditions (blue = VALID; black = INVALID). Grey mask represents time-slice of samples with significant results.

We explored differences in the cortical ERF responses to different logical reasoning modalities. Cortical amplitudes were time-locked compared for stimuli onset in the time-window from 250 to 800 ms after stimulus display. Whole time-window were statistically compared between conditions using a Cluster Based Permutation Test (CBPT) approach^40^, finding significant differences within 310 ms and 780 ms. Figure 3 shows the distribution of the significant cluster. In order to ease the interpretation of spatial and temporal relevance of such differences, we represented the cluster distribution in different temporal slices. Figures 4, 5, 6 and 7 represent the distribution of cortical dynamics throughout successive times-slices of 150 ms length (grey mask), with a 25 ms overlap. Within each slice, we highlighted the sensors which contributed to significant cluster. Topographical maps of amplitudes are individually displayed for each task condition and its difference (Figs. 4, 5, 6 and 7A), together with significant results distribution in each time-slice (Figs. 4, 5, 6 and 7B) and times-series (Figs. 4, 5, 6 and 7C).

Results showed a significant cluster with higher field negativity for the INVALID condition compared to the VALID condition (p = 0.0004). This “negative” cluster encompassed sensors located in right and middle frontal areas, with significant activity within the time window of 310 ms to 780 ms. Figures 4, 5, 6 and 7. B2 show the cluster dynamical distribution in successive time slices. Additionally, we found a significant cluster of higher field positivity for the INVALID condition, formed by left prefrontal sensors (p = 0.0259) within the time window of 430 ms to 670 ms. Figures 4, 5, 6 and 7. B1 displays the cluster distribution throughout time slices. The cortical source activity measured by sensor-space MEG ERF must be interpreted as a magnetic dipole localized between the higher positive and negative fields. In this sense, throughout the significant time window, main clusters exhibit certain variations in size and sensor distribution. At early stages of the significant time window (250–450 ms) (Figs. 4, 5A,B), a negative field cluster may be formed by two potential dipoles: one is located in left-middle prefrontal regions, with its highest positivity at left prefrontal sensors. The second one appears to be located in right parietal sources, with its highest positivity at right occipito-parietal sensors. However, this second dipole did not show a significant positive field cluster (p = 0.3). The later time window (450–800 ms) (Figs. 6, 7A,B), exhibits a well-localized source of activity in left-middle prefrontal regions, between both positive and negative significant clusters. However, it does not exhibit previous right occipito-parietal positivity or a parietal dipole, while presenting a potential secondary dipole with a non-significative positive cluster in right temporal regions beyond 650 ms. Thus, the more probable origin of this significant activity might be a magnetic dipole situated in left-medial parts of the prefrontal cortex, or anterior cingulate cortex (ACC), particularly within a temporal window of 400 to 780 ms (see B component in Figs. 4, 5, 6 and 7). The temporal and spatial distribution of amplitude differences are in accordance with classical electromagnetic components P300b, N400 and P600, whose neurocognitive implications will be discussed below.

## Discussion

This work has tried to determine if validity (systematic preservation of truth) leaves any trait in brain processing. The neuroelectrical MEG study compares inferences with the same content (same stimuli and same relational variables) but distinct logical complexity, showing that valid deductions leave an electric trail measurably distinct from logically invalid inferences. Both logically valid and invalid conditions show different amplitudes around P3, N4 and P6 electromagnetic components, including distribution sensors with significant and growing amplitude differences which reach a peak at 0.5–0.7 s. The spatial distribution of the significant electrical activity displayed in the MEG was remarkably the same in both conditions, located in the medial left prefrontal cortex and the ACC. Remarkably, in the logically valid condition, the amplitude and intensity of the electrical brain activity was significantly lower while the reaction time was 54.37% higher than in the logically invalid condition. The recursive computational nature of logically valid deductive inferences can be suggested as an explanation of this hypoactivity and retardment. Deductions in the valid condition result from recursive, explicit and monitored computations which are not only slower but also less demanding.

Amplitude differences between the conditions should be attributed to the processing of logical complexity, since the paradigm grants that besides logical complexity, both conditions have the same variables. These differences begin to be significant after 350 ms (negative cluster, see Fig. 4) or 420 ms (positive cluster, see Fig. 4), coinciding with the presence of a P300 potential that the neuroscientific literature^41–43^, and particularly the neuroelectric literature^21,22,45^, associates with premise integration^8,17–20,22–24^. The potential is found in both conditions, which is expected since both are fully integrable. In fact, in the positive cluster (see Fig. 4B), there are still no significant traces of valid deduction. The topology of this positive cluster confirms the activation of semantical left parietal brain areas related to integration and the P3B potential, confirming previous studies^7,9,17–20^ which suggested that semantical content determines the integration of premises. On the other hand, differences between conditions are significant in the negative cluster (see Fig. 4B). The literature describes the recruitment of the right hemisphere when integration difficulties appear^1,22,23^ or in the presence of complex logical deductions^7,8^; but in this case these difficulties do not exist, since both conditions are equally integrable and neither is especially complex. Therefore, differences between conditions must be attributed to the diminution of electrical activity in the valid condition in the negative cluster. Figure 4C shows the ERF evolution and confirms this process. In the following phase of the initial window (Fig. 5), the hypoactivity of the valid condition extends to the positive cluster, and the differences between valid and invalid inferences progressively increase. However, the amplitude diminution of valid inferences does not alter the common electrical profile of components in both conditions, which is slightly retarded in the valid condition. In the temporal window at 375–525 ms, a clear N400 component is found in both conditions (see Fig. 5B). The neuroelectrical literature on deduction has sometimes interpreted this wave not as an N400, but as an additional component to integration^24,43,44^, since the role of semantic content (processed by N400) is a disputed issue in formally valid deductions^23,24,45,46^ have also found evidence of N400-like deflations in deductive contexts, both in linguistical and visual settings. Its presence in the valid condition seems to show that semantic content processing accompanies but does not conflate with logical complexity.

In the initial phase of the late temporal window (500–650 ms) (see Fig. 6) the maximal differences between conditions is reached coinciding with the positive deflation in the valid condition at about 550 ms, which may be interpreted as an advanced P600 potential. To explain these differences, we can exclude attributing the P600 component to integration difficulties^20,21^ or syntactic rule violation^47^, or even pragmatic maxims violations, since both conditions share the same contents. Consequently, these differences should be attributed to the processing of logical complexity, not relational complexity (which does not differ between conditions). Moreover, this late potential has been extensively associated in the literature with reprocessing, which we may interpret deductively in this situation. Antecedents for this late deductive interpretation have been found in the logical interpretation of quantifiers^48^, in the processing of propositional connectives^13^ and after deductive training^15,16^.

It has been described how the intensity of the electrical brain activity progressively diminishes in the valid condition while simultaneously the reaction time significantly increases (see Table 1). What kind of physical process associated with valid deduction is automatic (less demanding) and temporally (neurally and conductually) slow? The most direct and plausible answer to this question is: a recursive or computational process. A theoretical argument and a practical argument ground this answer. From a theoretical viewpoint, a deductive system is one of the equivalent formal tool sat the origin of the contemporary notion of recursiveness or computability with its strengths and limitations^49^. Logically valid deductions are all provable or demonstrable by means of complete deductive formal systems, even if not all valid deductions are algorithmically decidable^5^. In this regard, valid deductions are computational in a literal sense, not in the metaphorical sense (in which many neural processes are said to compute). As it is well known, logically valid deductions combine a finite number of rules and axioms, and for this reason, they are automatable in different kinds of abstract and physical devices^49^. Moreover, automatable cortical processes are known to be electrically less demanding^50^, and therefore, the fact that logically valid deductions are less demanding is consistent with their recursive or computational nature.

The electrical hypoactivity in the valid condition is explained by the minor energy demand of valid deductions with respect to non-valid inferences. The automatic application of logical algorithms, such as those with elementary complexity employed in SET, seems to demand less effort and energy as has been observed in other cognitive^51^ and motor^52^ experiments. The automatization of valid deductive routines allows one to save energy without exposing an inference to systematic error.

Cerebral automatisms are typically swift and rapid^53^. How, then, can the remarkable retardment in valid deductive processing be explained? It can be explained by the recursive nature of valid inferences, which are deductive proofs of SET properties. Notice that recursive computational procedures are faster and more efficient than simple iterations^54^, but they are still slow compositional processes based on strict routines and they exclude abbreviated procedures or heuristic jumps. Logically valid deductive reasoning is a computational step-by-step procedure which is fully explicit and compositional. That is, subjects must first process the cards composing the items and then combine them and apply the definition of SET with its logical structure. This is a recursive task which consumes time as a linear function of its logical and relational complexity. For this reason, time-reactions are significantly slower in the valid condition, and neuroelectrically, significant differences appear after 600 ms (see). Recursion as a computational feature found in cerebral processes has been found in visual^54^ and motor^55^ processes which may incorporate implicit deductive tasks. In SET, recursive computations correspond to explicitly monitored step by step procedures or proofs which explain the slowness in the automatable processes. Logical training does not in general reduce the reaction time solving valid deductive reasoning^15,16^, because valid deductions don't allow heuristic shortenings and in fact logical training inhibits them. Previous studies on the logical inference of children^56^ and professional mathematicians^57^ have shown similar computational retardments also associated with recursive tasks. The paradigm does not allow us to determine which specific sequence or valid deductive rule is followed in the valid condition, but we know that some kind of valid ordered sequence of SET rules is successfully followed. On the other hand, the deductive monitorization of inference is also a slow recursive task in which standard heuristic responses are systematically avoided. Moreover, late hypoactivity and recursiveness in deductive reasoning is consistent with earlier neuroscientific^12^ and psychological^23^ research on deductive reasoning identifying inhibitory strategies to avoid both heuristic or random conclusions in logically valid deductive settings.

Therefore, the recursive features of the cortical processing of logically valid deduction explain the electrical and conductual results of the experiment, showing that logically valid inferences have factive electrical properties absent in non-valid inferences. Moreover, the recursiveness in precise mathematical models of brain activity will allow to verify or refute its presence in cerebral processes. Consequently, the question posed in the introduction: are there factual marks of valid deductions as time-consuming events? is answered affirmatively. There are electrical marks of a normative notion such as logical validity.

The results point to slow automatism as a factive feature that is present in the logically valid condition and not in the invalid condition. However, to fully characterize validity in factive terms, we should show that only valid inferences have those factive properties. We cannot exclude sporadic valid deductions within the non-valid condition, and moreover, we cannot associate them with less electrical demand or a longer temporal duration. Let us imagine a trial in the invalid condition in which a certain rule hypothesis is default. The agent validly rejects the hypothesis (for example via Modus Tollens). Let us call all logically valid deductive inferences non-explicitly produced in any inferential process (deductive or not) and only those inferences *microdeductions*. Microdeductions are logically valid deductions that happen within non-deductive and/or non-valid inferences. For example, uncompleted, unconscious or frustrated inferential processes may also contain microdeductions. A full electrical characterization of any kind of logically valid deductions should therefore include microdeductions. We formulate the conjecture that microdeductions are also recursive and less electrically demanding. This hypothesis is consistent with the recursive or computational nature of logically valid deductions in general but confirming or refuting the hypothesis requires a new experimental paradigm distinct from the protocol used in this work. Consequently, the results obtained in the experimental condition imply that all logically valid inferences in this condition are slow automatisms. If the microdeductive conjecture were confirmed, then sufficient physical (neuroelectrical) conditions for logical validity would be defined.

From a spatial point of view, the significant activity at the maximal distinction period between valid and invalid conditions (400–780 ms) have been located in the magnetic dipole situated in left medial parts of the prefrontal cortex, or anterior cingulate cortex (ACC) (see B components in Figs. 4, 5, 6, and 7). Both deductive conditions presented the same spatial origin, irrespective of their validity. On the other hand, valid deductive activity is a notable example of a higher cognitive process requiring top-down control, which is exactly what is attributed to the ACC by the literature^58^. Valid deductive abilities are excellent candidates for verifying maintenance, inhibition and monitorization processes recruited by the ACC. This magnetic dipole is consistent with one of the deductive “core” areas identified by Monti and Oshershon^24^, Monti^25,26^ and Monti and Coetzee^27–29^. A minoritarian but recalcitrant series of studies with distinct techniques (fMRI, PET, and now MEG) seem to show that part of the cerebral processing of logical complexity happens in an area independent from the brain’s language areas. Other electrical studies of deduction^8,19,21,22^ tend to understand it as a consequence of content integration of premises. To deal with this disagreement, the results in this study suggest that all valid deductive inferences are recursive and hypoactive. Under this general pattern, one can consistently explain the presence of three neural profiles for valid deductive inferences in the literature: (i) those that result from logical complexity computations (slow and late (P6) cognitive events), (ii) those that result from content integration (P3 deductions) and (iii) those that are even faster than awareness such as Modus Ponens (below 50 ms according to Reverberi’s results^59^. Temporal and spatial results show that late deductions are monitored and reprocessed, while earlier deductions are probably not. In this regard, there is no blatant contradiction between the majoritarian semantical understanding of deductions as content-dependent and the minoritarian logical understanding of deductions as recursive.

As a conclusion, *deductions* can be considered to be adventitious inferential processes proceeding with purely heuristic rules. Probably, most inferences that the human brain helps to produce are of this type. But electrical brain activity in the valid condition shows that there are non-adventitious deductions which are recursive, automatable and valid. This study has taken advantage of MEG’s high resolution images to uncover the necessary electrical conditions for valid deductions, even if sufficient conditions have only been conjectured and are the objective of future research confirming or refuting the microdeduction hypothesis.

## Data Availability

Raw data are available in openneuro: https://doi.org/10.18112/openneuro.ds003483.v1.0.0.

## References

[1] Goel, V. Hemispheric asymmetry in the prefrontal cortex for complex cognition. in *Handbook of Clinical Neurology*. 179–96. (Elsevier, 2019). 10.1016/B978-0-12-804281-6.00010-0 31590729

[2] Harman G (1984). Logic and Reasoning Foundations: Logic, Language, and Mathematics.

[3] Evans JSBT, Over DE (2013). Reasoning to and from belief: Deduction and induction are still distinct. Think Reason..

[4] Oaksford M (2015). Imaging deductive reasoning and the new paradigm. Front. Hum. Neurosci..

[5] Boolos G, Burgess J, Jeffrey R (2007). Computability and Logic.

[6] Kleiter G (2018). Adam's p-validity in the research on human reasoning. J. Appl. Logics..

[7] Goel V (2007). Anatomy of deductive reasoning. Trends Cogn. Sci..

[8] Prado J, Der Henst J-B, Van NIA (2010). Recomposing a fragmented literature: How conditional and relational arguments engage different neural systems for deductive reasoning. Neuroimage.

[9] Prado J, Chadha A, Booth JR (2011). The brain network for deductive reasoning: A quantitative meta-analysis of 28 neuroimaging studies. J. Cogn. Neurosci..

[10] Prado J (2018). The relationship between deductive reasoning and the syntax of language in Broca's area: A review of the neuroimaging literature. L'annee Psychol..

[11] Brunetti R, Indraccolo A, Mastroberardino S, Spence C, Santangelo V (2017). The impact of cross-modal correspondences on working memory performance. J. Exp. Psychol. Hum. Percept. Perform..

[12] Parsons LM, Monti MM, Martinez MJ, Osherson D (2005). Neural correlates of deductive inference: A language-independent distributed network. Acta Neurobiol. Exp..

[13] Baggio G, Cherubini P, Pischedda D, Blumenthal A, Haynes JD, Reverberi C (2016). Multiple neural representations of elementary logical connectives. Neuroimage.

[14] Houdé O, Borst G (2014). Measuring inhibitory control in children and adults: Brain imaging and mental chronometry. Front. Psychol..

[15] Mackey AP, Miller Singley AT, Bunge SA (2013). Intensive reasoning training alters patterns of brain connectivity at rest. J. Neurosci..

[16] Álvarez Merino P, Requena C, Salto F (2018). Evidence linking brain activity modulation to age and to deductive training. Neural Plast..

[17] Reverberi C, Cherubini P, Rapisarda A, Rigamonti E, Caltagirone C, Frackowiak RSJ, Paulescu E (2007). Neural basis generation of conclusions in elementary deduction. Neuroimage.

[18] Reverberi C, Shallice T, D’Agostini S, Skrap M, Bonatti LL (2009). Cortical bases of elementary deductive reasoning: Inference, memory, and metadeduction. Neuropsychologia.

[19] Reverberi C, Cherubini P, Frackowiak RSJ, Caltagirone C, Paulesu E, Macaluso E (2010). Conditional and syllogistic deductive tasks dissociate functionally during premise integration. Hum. Brain Mapp..

[20] Reverberi C, Bonatti LL, Frackowiak RSJ, Paulesu E, Cherubini P, Macaluso E (2012). Large scale brain activations predict reasoning profiles. Neuroimage.

[21] Prado J, Kaliuzhna M, Cheylus A, Noveck IA (2008). Overcoming perceptual features in logical reasoning: An event-related potentials study. Neuropsychologia.

[22] Bonnefond M, Van der Henst JB (2009). What’s behind an inference? An EEG study with conditional arguments. Neuropsychologia.

[23] Bonnefond M, Van der Henst J-B (2013). Deduction electrified: ERPs elicited by the processing of words in conditional arguments. Brain Lang..

[24] Bonnefond M, Noveck I, Baillet S, Cheylus A, Delpuech C, Bertrand O (2013). What MEG can reveal about inference making: The case of ifthen sentences. Hum. Brain Mapp..

[25] Beckmann JF, Birney DP, Goode N (2017). Beyond psychometrics: The difference between difficult problem solving and complex problem solving. Front. Psychol..

[26] Evans JSBT, Thompson VA, Over DE (2015). Uncertain deduction and conditional reasoning. Front. Psychol..

[27] Monti MM, Parsons LM, Osherson DN (2009). The boundaries of language and thought in deductive inference. Proc. Natl. Acad. Sci. USA..

[28] Parsons LM, Osherson D (2001). New evidence for distinct right and left brain systems for deductive versus probabilistic reasoning. Cereb. Cortex..

[29] Monti MM, Osherson DN, Martinez MJ, Parsons LM (2007). Functional neuroanatomy of deductive inference: A language-independent distributed network. Neuroimage.

[30] Holyoak M, Monti MM (2020). Relational integration in the human brain: review and synthesis. J. Cogn. Neurosci..

[31] Coetzee J, Monti M, Iacoboni M, Wu A, Johnson M (2019). Separability of logic and language: A TMS study. Brain Stimul. Basic Transl. Clin. Res. Neuromodulation..

[32] Coetzee JP, Monti MM (2018). At the core of reasoning: Dissociating deductive and non-deductive load. Hum. Brain Mapp..

[33] Rips L (1994). The Psychology of Proof: Deductive Reasoning in Human Thinking.

[34] Wilhelm O (2005). Measuring Reasoning Ability Handbook of Understanding and Measuring Intelligence.

[35] Rotello CM, Heit E (2009). Modeling the effects of argument length and validity on inductive and deductive reasoning. J. Exp. Psychol. Learn. Mem. Cogn..

[36] Heit E, Rotello CM (2014). Traditional difference-score analyses of reasoning are flawed. Cognition.

[37] Merino PÁ, Requena Hernández C, Alemany FS (2020). The measurement of factive deductivity: A psychological and cerebral review. Reason Games Cogn. Logic..

[38] Adams, E. *A primer on Probability Logic*. (CSLI publ., 2016 [1996]). ISBN: *157586066X*

[39] Taulu S, Simona J (2006). Spatiotemporal signal space separation method for rejecting nearby interference in MEG measurements. Phys. Med. Biol..

[40] Oostenveld R., Fries, P., Maris, E., & Schoffelen, JM. FieldTrip: Open source software for advanced analysis of MEG, EEG and invasive electrophysiological data. *Comput. Intell. Neurosci.* (2011). 10.1155/2011/156869 PMC3021840 21253357

[41] Belouchrani A, Abed-Meraim K, Cardoso JF, Moulines E (1997). A blind source separation technique using second order statistics. IEEE Trans. Signal Process..

[42] Lei Y, Liang X, Lin C (2017). How do the hierarchical levels of premises affect category-based induction: Diverging effects from the P300 and N400. Sci. Rep..

[43] Baggio G, van Lambalgen M, Hagoort P (2015). Logic as Marr's computational level: Four case studies. Top. Cogn. Sci..

[44] Fangmaier T, Knauff M, Ruff C, Sloutsky V (2006). fMRI evidence for a three-stage model of deductive reasoning. J. Cogn. Neurosci..

[45] Alvarez-Merino P, Requena C, Salto F (2019). Brain localization of semantic processing. Rev. Neurol..

[46] Maddox, C.B. *An Electroencephalogram Investigation of Two Modes of Reasoning*. Doctoral Dissertation, Columbia University (2012).

[47] Regel S, Meyer L, Gunter TC (2014). Distinguishing neurocognitive processes reflected by P600 effects: Evidence from ERPs and neural oscillations. PLoS ONE.

[48] Politzer-Ahles S, Gwilliams L (2015). Involvement of prefrontal cortex in scalar implicatures: Evidence from magnetoencephalography. Lang. Cogn. Neurosci..

[49] Epstein RL, Carnielli WA (2008). Computability. Computable Functions, Logic and the Foundations of Mathematics.

[50] Westbrook A, Braver TS (2015). Cognitive effort: A neuroeconomic approach. Cogn. Affect. Behav. Neurosci..

[51] Zhen Y, Li D, Ding R, Huang Z, Qu Z, Ding Y (2018). Automatic detection of orientation contrast occurs at early but not earliest stages of visual cortical processing in humans. Front. Hum. Neurosci..

[52] Mars RB, Coles MGH, Hulstijn W, Toni I (2013). Delay-related cerebral activity and motor preparation. Cortex.

[53] Schneider W, Chein JM (2003). Controlled & automatic processing: Behavior, theory, and biological mechanisms. Cogn. Sci..

[54] Al Roumi, F., Marti, S., Wang, L., Amalric, M. & Dehaene, S. An abstract language of thought for spatial sequences in humans. *bioRxiv* (2020).

[55] Martins MJ, Bianco R, Sammler D, Villringer A (2019). Recursion in action: And fMRI study on the generation of new hierarchical levels in motor sequences. Hum. Brain Mapp..

[56] Schwartz F, Epinat-Duclos J, Léone J, Prado J (2017). The neural development of conditional reasoning in children: Different mechanisms for assessing the logical validity and likelihood of conclusions. Neuroimage.

[57] Amalric M, Dehaene S (2016). Origins of the brain networks for advanced mathematics in expert mathematicians. Proc. Natl. Acad. Sci..

[58] Dosenbach NUF, Fair DA, Cohen AL, Schlaggar BL, Petersen SE (2008). A dual-networks architecture of top-down control. Trends Cogn Sci..

[59] Reverberi C, Pischedda D, Burigo M, Cherubini P (2012). Deduction without awareness. Acta Psychol..

